# The double-edged nature of trained immunity

**DOI:** 10.7554/eLife.107787

**Published:** 2025-07-03

**Authors:** Chrissy M Leopold Wager, Larry S Schlesinger

**Affiliations:** 1 Host Pathogen Interactions Program, Texas Biomedical Research Institute San Antonio United States

**Keywords:** trained immunity, acute lung injury, beta-glucan, alveolar macrophage, Mouse

## Abstract

Trained immunity in alveolar macrophages can lead to damaging lung inflammation, confirming the importance of context in this phenomenon.

**Related research article** Prével R, Pernet E, Tran KA, Sadek A, Sadeghi M, Lapshina E, Jurado L, Kristof AS, Moumni M, Poschmann J, Divangahi M. 2024. β-glucan reprograms alveolar macrophages via neutrophil/IFNγ axis to promote lung injury. *eLife* **13**:RP102068. doi: 10.7554/eLife.102068.

The concept of trained immunity has transformed our understanding of how the innate immune system responds to stimuli. For many years, the responses of innate immune cells – such as monocytes, macrophages, natural killer cells and neutrophils – were seen as short-lived and non-specific. However, we know now that these immune cells can adapt their responses after being exposed to a stimulus for the first time. This initial encounter trains these cells to respond more vigorously to subsequent challenges, which typically leads to increased resistance to various infections. This memory-like state is typically triggered by microbial components like β-glucan, the BCG vaccine, and certain endogenous signals (Netea et al., 2020). The training that follows these exposures results in long-term changes in transcription, chromatin structure and cellular metabolism in the immune cells. However, while trained immunity is often protective, it can also carry costs.

Deep in the lungs, for example, cells called alveolar macrophages are constantly bombarded with inhaled chemicals, pathogens and particulates, and these cells play a crucial role in balancing immune responses while keeping inflammation at bay. However, molecules known as lipopolysaccharides, found in the membranes of certain bacteria, can lead to excessive inflammation, resulting in acute lung injury. The relationship between trained immunity and inflammation has so far remained unclear.

Now, in eLife, Maziar Divangahi of McGill University and colleagues – including Renaud Prével as first author – report more insights into the double-edged nature of trained immunity in the lungs (Prével et al., 2024). The researchers investigated whether β-glucan can induce trained immunity in alveolar macrophages in mice, and how this affects acute lung injury.

Mice were injected with β-glucan and, seven days later, were treated with bacterial lipopolysaccharide or an immunostimulant called poly(I:C) to simulate viral infections. Mice that received bacterial lipopolysaccharide exhibited an increased number of neutrophils in the lungs. Furthermore, they developed elevated levels of several inflammatory chemical messengers, such as TNF-α, IL-6 and CXCL1, and displayed signs of acute lung injury (Figure 1). Mice treated with the immunostimulant also demonstrated a rise in neutrophils and chemical messengers, apart from TNF-α, which remained unchanged.

**Figure 1.**
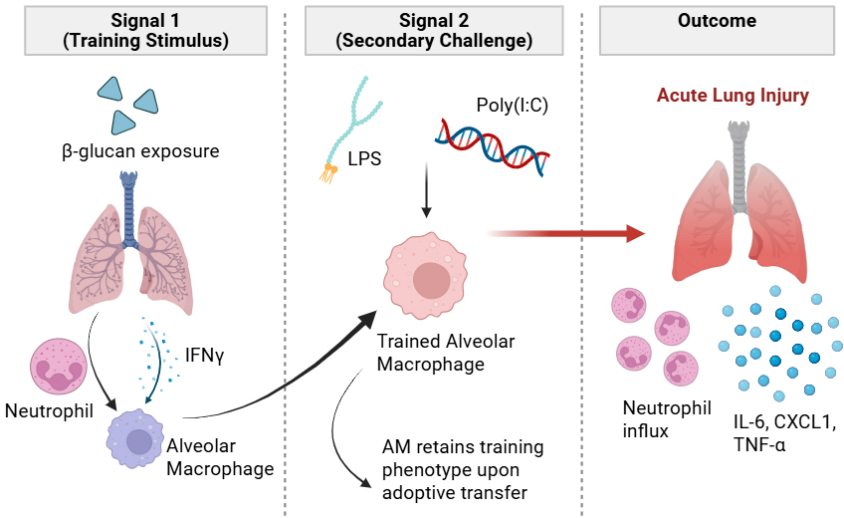
Trained immunity and acute lung injury. Injecting mice with β-glucan (Signal 1) resulted in trained immunity for alveolar macrophages (left panel). This trained immunity depended on neutrophils and type II interferon signalling (IFNγ). The macrophages remained in their trained state when transferred into mice that lacked immune cells (middle panel). Exposure to a secondary challenge (Signal 2) – such as a bacterial stimulus (LPS) or a viral stimulus (poly(I:C)) – later re-engaged the trained macrophages and induced a strong inflammatory response. This response includes neutrophil influx and increased production of several inflammatory chemical messengers (IL-6, CXCL1 and TNF- α), which led to acute lung injury (right panel). LPS: lipopolysaccharides; poly(I:C): polyinosinic:polycytidylic acid; AM: alveolar macrophage; IL-6: interleukin 6; CXCL1: chemokine (C-X-C motif) ligand 1; TNF-α: tumor necrosis factor alpha. This figure was created with BioRender.com.

The work of Prével et al. is consistent with studies that have reported that β-glucan aggravates disease in models of periodontitis and arthritis (Haacke et al., 2025). However, this contrasts with previous work, which showed that training with β-glucan can reduce lung fibrosis caused by exposure to the chemotherapy drug, bleomycin (Kang et al., 2024). These conflicting findings emphasize the need to understand the contexts in which trained immunity may backfire.

Prével et al. – who are based at McGill University and institutes in Morocco and France – confirmed that alveolar macrophages are key mediators of the exaggerated immune response. Following β-glucan exposure, the number of immune cells in the lungs remained unchanged; however, the macrophages underwent metabolic and transcriptional reprogramming, which led to an increase in the production of inflammatory cytokines. This suggests that functional changes in the macrophages, rather than an increase in cell number, leads to stronger inflammation. Furthermore, transplanting trained alveolar macrophages into mice lacking these cells, followed by administration of lipopolysaccharide, initiated a heightened inflammatory response. This demonstrated that the phenotype is intrinsic to alveolar macrophages and independent of circulating monocytes.

Prével et al. also found that β-glucan training required type II interferon signaling but not type I. This contrasts with previous work showing a key role for the latter in lipopolysaccharide-trained alveolar macrophages exposed to pneumococcal bacteria (Zahalka et al., 2022). Indeed, type II interferon signaling has also been implicated in training macrophages in response to bacterial, fungal and viral infections (Leopold Wager et al., 2018; Tran et al., 2024; Wang et al., 2023; Arafa et al., 2022).

Based on these results, Prével et al. go on to propose a framework in which the nature of the initial training signal (signal 1) and the nature of the secondary challenge (signal 2) determine the immunological outcome of trained immunity (Figure 1). This framework implies that trained immunity is not defined solely by the training signal, but also by the context in which the cells are re-engaged.

Other variables may also influence the outcomes of trained immunity, including differences in laboratory readouts (e.g., cytokines, tissue damage, microbial clearance) and routes of administration. For example, intravenous but not subcutaneous administration of the BCG vaccine enhanced antiviral responses of alveolar macrophages (Tran et al., 2024).

Perhaps most importantly, the source and environment of the responding immune cells matter greatly. Much research has focused on blood-derived monocytes and their progenitors, including hematopoietic stem cells (Moorlag et al., 2020; Kaufmann et al., 2018; Netea et al., 2020). However, resident macrophages in tissues like the lung are increasingly recognized as key players in local trained immunity (Tran et al., 2024; Zahalka et al., 2022; Chakraborty et al., 2023; Prével et al., 2024). Moreover, neighboring immune cells can also shape trained immunity, as shown by the fact that neutrophils are required for the training of alveolar macrophages in the current study.

The study of Prével et al. is particularly compelling because, in addition to being found in the cell walls of bacteria, β-glucan is also a ubiquitous component of fungal cell walls, many of which reside within our microbiota. This raises the possibility that alveolar macrophages are constantly trained by our unique environments, lifestyles and even age.

The developmental history of alveolar macrophages adds another layer of complexity. Resident alveolar macrophages arise from fetal monocytes and self-renew, but during infection or injury, they can be replaced by monocyte-derived alveolar macrophages. These recruited cells are often more inflammatory, which helps to fight pathogens but also carries a higher risk of tissue damage. Future studies to assess how β-glucan training affects the development of the lung macrophage population will be of interest as well as the durability and molecular basis of the trained state.

Ultimately, the work of Prével et al. offers a nuanced view of trained immunity in an organ where immune restraint is often more important than immune reactivity. Understanding how trained immunity is shaped by training and cellular origin, signaling molecules, and the environment will be essential. As we continue to explore how innate immune memory is generated and maintained, one thing is clear: context is everything.
